# Lecanemab induces phenotypic change in glial and microglial cells in Alzheimer’s disease models: vitro and vivo evidences

**DOI:** 10.1002/alz.094860

**Published:** 2025-01-09

**Authors:** Noelle Callizot, Alexandre Henriques, Steidl Esther, Laura Rouviere, Bilal El Waly, Alexandre Wojcinski, Philippe Poindron

**Affiliations:** ^1^ Neuro‐Sys, Gardanne France

## Abstract

**Background:**

Alzheimer’s disease (AD) is characterized by the extracellular accumulation of senile plaques composed of beta‐amyloid (Aβ) and the intracellular deposition of neurofibrillary tangles composed of hyperphosphorylated tau (Tp). Aβ induces a chronic neuroinflammation that contributes to the neurite network disorganization and finally neuronal death.

Lecanemab, a humanized monoclonal antibody (Ab) that recognizes protofibrils/oligomers and prevents Aβ deposition, is the first US approved Ab for AD. Its mode of action especially on inflammatory cells is still vague. We investigated Lecanemab’s effects in vitro in microglial cells and astrocytes phenotype after Aβ stress and in an aged mouse brains exposed to Aβ1‐42 (model of AD) and treated with Lecanemab

**Method:**

Primary rat cortical neurons, co‐cultured with microglia and astroglial cells were used. After 11 days of culture, cells were injured with Aβ up to 72h. Lecanemab was applied one hour before Aβ injury. After fixation, microglia and astroglia phenotype were analyzed. For both cell types, pro and anti‐inflammatory markers were assessed. Aged mice were stereotaxically infused with Aβ oligomeric fraction in CA1 hippocampal area. Animals were chronically treated with Lecanemab up to 4 weeks after the lesion. Histological analysis on brains was performed, inflammation markers were investigated.

**Result:**

Application of Aβ induced a proliferation of astrocytes and microglial cells. In addition, a significant increase in M1 microglia was observed (TREM2/OX‐41 and Iba1) 72h after Aβ application followed with an increase of phagocytosis phenotype. A strong reduction of CD206 (M2 markers) was also detected. For astrocytes, a moderate astrogliosis was observed. Interestingly, Lecanemab treatment was able to increase number of S100A (+) cells (A2 population), associated with a moderate increase of GFAP area. In AD aged mice, cognition performances were improved and were associated with a significant reduction of brain amyloidic load in treated animals. Hippocampal section showed an increase of microglial activation (M1 and M2), and large astrogliosis.

**Conclusion:**

Lecanemab was able to decrease Aβ1‐42‐induced toxicity and modulated the Aβ1‐42‐induced neuroinflammatory response. These important and intriguing changes were observed both in vitro and in vivo. This study attempted to decipher the complex and unclear mode of action of Lecanemab.

